# The Pulmonary Manifestation of Mastocytosis: Experiences of the National Reference Centre of Excellence

**DOI:** 10.3390/jcm14155455

**Published:** 2025-08-03

**Authors:** Marlena Sztormowska, Aleksandra Górska, Maciej Piskunowicz, Lucyna Górska, Wojciech Nazar, Marta Chełmińska, Krzysztof Kuziemski, Ewa Jassem, Marek Niedoszytko

**Affiliations:** 1 Department of Allergology, Medical University of Gdansk, Smoluchowskiego 17, 80-214 Gdańsk, Poland; marlena.sztormowska@gumed.edu.pl (M.S.); aleksandra.gorska@gumed.edu.pl (A.G.); lucyna.gorska@gumed.edu.pl (L.G.); marta.chelminska@gumed.edu.pl (M.C.); mnied@gumed.edu.pl (M.N.); 2 Department of Radiology, Medical University of Gdansk, Smoluchowskiego 17, 80-214 Gdańsk, Poland; maciej.piskunowicz@gumed.edu.pl; 3 Department of Pulmonology, Medical University of Gdansk, Smoluchowskiego 17, 80-214 Gdańsk, Poland; krzysztof.kuziemski@gumed.edu.pl (K.K.); ewa.jassem@gumed.edu.pl (E.J.)

**Keywords:** blood–air barrier, interstitial lung diseases, mast cell activation syndrome, mastocytosis, pulmonary diffusing capacity, spirometry

## Abstract

**Background:** Patients with mastocytosis may present with exacerbated respiratory symptoms and lung diseases resulting from mast cell mediator release. However, their prevalence and severity level remain under debate. The study aims to analyze the prevalence of respiratory symptoms and the usefulness of lung function tests like spirometry, diffusing capacity of the lung for carbon monoxide (DLCO), and high-resolution computed tomography (HRCT) of the chest in mastocytosis patients presenting with dyspnea, cough, and exercise intolerance. **Methods:** We included 104 patients with mastocytosis and 71 healthy controls. Data collection encompassed patient interview, clinical examination, spirometry, DLCO, and chest HRCT. Diagnosis of mastocytosis included bone marrow biopsies and serum tryptase measurements. **Results:** Compared to controls, patients with mastocytosis exhibited significantly lower values in FEV1/VC ratio, absolute DLCO/VA, predicted DLCO/VA, absolute DLCOcSB, and predicted DLCOcSB (*p* < 0.001). Commonly reported respiratory symptoms included dyspnea (36.5%), chest tightness (22.1%), and wheezing (9.6%). Airway obstruction was identified in 7.7% of patients; however, it appeared to be independent of the mastocytosis subtype. A decreased DLCO/VA ratio was observed in 4.8% of patients, but HRCT did not reveal any evidence of underlying lung disease. **Conclusions:** Mastocytosis appears to be a risk factor for the occurrence and exacerbation of respiratory symptoms. However, airway obstruction and impairment of the alveolar–capillary membrane seem to occur independently of the clinical subtype of mastocytosis. Additionally, the causal relationship between pulmonary involvement, mast cell infiltration of the alveolar–capillary membrane, and the systemic circulation of mast cell mediators remains unclear and requires further research.

## 1. Introduction

Mastocytosis is a group of myeloproliferative diseases characterized by excessive growth of mast cells and their accumulation in various organs and tissues, including the bone marrow, skin, liver, spleen, lymph nodes, and gastrointestinal tract [1,2,3,4]. Pulmonary manifestation is infrequent; in most cases, histopathological confirmation is not performed [1,2,3,4]. However, there are frequent symptoms whose etiology may result from the involvement of the respiratory system, such as dyspnea and impaired exercise tolerance, as well as symptoms of respiratory system involvement during the release of mediators, such as cough and chronic fatigue [2,5,6].

Patients with symptomatic mastocytosis are predisposed to asthma-like bronchial hyper-responsiveness due to airway mast cell infiltration and the release of histamine, prostaglandin D2, and tryptase [7,8]. Therefore, in patients with systemic mastocytosis (SM), it is recommended to exclude bronchial hyper-responsiveness [8,9]. There were several cases of respiratory involvement (such as interstitial disease) in patients with aggressive forms of mastocytosis [5,7,8,9]. Respiratory function tests showed reduced carbon monoxide diffusion capacity, and chest X-rays revealed fibrosis and pleural effusion [5,6,7,8,9]. However, in most cases, biopsy of the lesions was not performed [5,6,7,8,9].

In patients with mastocytosis, high-resolution computed tomography (HRCT) sometimes reveals an area of ground-glass opacity and widening of interalveolar septa, as well as micronodular and cystic changes and enlargement of lymph nodes [7,10]. In addition, the presence of atypical mast cells (CD117(+), CD34(−), CD2(−), CD25(+)) was found in bronchoalveolar lavage, lung biopsies, and lymph nodes [5,7,11].

Mast cells are physiologically present in healthy human lung tissue, with the highest concentrations located in the lung parenchyma compared to the bronchi and bronchioles [12]. They are notably enriched in the intimal layer of pulmonary blood vessels and surrounding perivascular spaces [13,14,15]. In healthy lungs, mast cells contribute significantly to tissue homeostasis by coordinating innate and adaptive immune responses to respiratory pathogens and facilitating normal wound repair following injury [12,13,14,15].

In pathological settings, chronic or inappropriate activation of mast cells is a hallmark of several inflammatory lung diseases [12,13,14,15]. In asthma, there is compelling evidence that mast cell mediators—such as histamine, tryptase, chymase, cytokines, and leukotrienes—induce bronchoconstriction, mucus hypersecretion, vascular permeability, and recruitment of eosinophils and neutrophils, contributing to both inflammation and airway remodeling [12,13,14,15].

Emerging studies also implicate mast cells in chronic obstructive pulmonary disease (COPD) and idiopathic pulmonary fibrosis (IPF). In COPD and IPF lungs, there is a marked upregulation of carboxypeptidase A3 (CPA3) expression in mast cells, which correlates with structural pathology and impaired lung function [16]. The density of MCTC-type mast cells (expressing both tryptase and chymase) and their expression of TGF-β strongly correlate with fibrosis severity, fibroblast foci formation, and reduced pulmonary function metrics [17].

Furthermore, mast cells actively contribute to pulmonary vascular remodeling in experimental pulmonary hypertension (PH) models [18]. Mast cell-deficient animals develop less vascular remodeling and right ventricular hypertrophy when exposed to hypoxia or monocrotaline, while pharmacological stabilizers of mast cells dampen PH development [15,18]. In limited human trials, mast cell inhibitors like cromolyn and fexofenadine reduced circulating angiogenic markers and pulmonary vascular resistance [13,14,15,18].

Other inflammatory or fibrotic pulmonary conditions—including acute respiratory distress syndrome (ARDS) and lung cancer—also show evidence of mast cell involvement [12,15,19]. Mast cell activation and mediator release have been documented in ARDS and COVID-19-associated lung injury, while in some lung tumors, released TNF-α and other mediators may influence tumor behavior and the tumor microenvironment [12,15,19].

The blood–air barrier plays a critical role in mediating gas exchange between the alveoli and pulmonary capillaries. Its functional integrity is essential for effective oxygen and carbon dioxide diffusion [20,21,22]. Pathological changes such as thickening of the alveolar–capillary membrane, a reduction in the effective surface area for gas exchange, ventilation–perfusion mismatch, or anemia can significantly impair pulmonary diffusion capacity. These abnormalities are characteristic of interstitial lung diseases, including pulmonary fibrosis, sarcoidosis, and congestive heart failure.

Overall, patients with mastocytosis may exhibit exacerbated respiratory symptoms and an increased susceptibility to pulmonary diseases due to the release of mast cell mediators. However, the prevalence, severity, and underlying pathophysiological mechanisms of these manifestations remain subjects of ongoing investigation and debate.

### Aim

Thus, the aim of the study was to analyze the prevalence of respiratory symptoms and the usefulness of lung function tests like spirometry, diffusing capacity of the lung for carbon monoxide (DLCO), and HRCT of the chest in mastocytosis patients presenting with dyspnea, cough, and exercise intolerance.

## 2. Materials and Methods

This retrospective, cross-sectional study was performed according to the STrengthening the Reporting of OBservational studies in Epidemiology (STROBE) Statement [23].

A total of 104 Caucasian patients with mastocytosis and mast cell activation syndrome from the Department of Allergology, Medical University of Gdansk, were studied (median age 46 years; 95% CI 44.0–49.0; 34.6% males).

The control group consisted of 71 healthy persons (median age 39 years; 95% CI 38.2–45.0; 50.7% males).

### 2.1. Inclusion and Exclusion Criteria

The primary inclusion criterion for the patient group was a confirmed diagnosis of systemic or cutaneous mastocytosis or mast cell activation syndrome (MCAS) in accordance with the most recent guidelines [2,3].

The following exclusion criteria were applied: contraindications to spirometry (e.g., aneurysm, recent ophthalmic surgery, increased intracranial pressure, hemoptysis of unknown origin, pneumothorax, recent myocardial infarction, or recent stroke); pregnancy or breastfeeding; acute respiratory infection; or any clinically significant medical condition that, in the investigator’s opinion, could interfere with adherence to the study protocol or the subject’s ability to provide informed consent [24,25].

Healthy controls had never smoked and denied taking medications resulting in side effects in the lung tissue, and they had never been exposed to damaging occupational factors. Furthermore, none of them had a history of cardiac diseases or diabetes.

### 2.2. Study Design

Both patients and healthy controls underwent routine medical interviews and physical examinations. Each patient was evaluated by a board-certified clinical allergologist. During the medical review, the physician inquired about any symptoms suggestive of mast cell activation.

To objectively assess dyspnea, a modified Medical Research Council (mMRC) scale was used [26]. Physical examination included chest auscultation, blood pressure measurement, and heart rate assessment. Subsequently, blood samples were collected for laboratory analyses, including complete blood count and troponin I concentration.

All patients underwent bone marrow examination, including histopathological, cytological, and flow cytometric evaluation (CD2, CD25), as well as baseline serum tryptase (sBT) measurement, in accordance with WHO standards. Serum tryptase levels were determined using the ImmunoCAP Tryptase assay (manufacturer). The upper normal reference value for sBT was 11.4 ng/mL. The KIT D816V mutation was identified using quantitative polymerase chain reaction (qPCR).

Cutaneous involvement was assessed through clinical inspection and confirmed by skin biopsy. Immunohistochemical staining for tryptase or CD117 (anti-KIT) was used to visualize mast cell infiltration.

### 2.3. Systemic and Cutaneous Mastocytosis–Diagnostic Criteria

The diagnosis of SM was established according to the latest International Consensus Classification/World Health Organization classification systems [3,27].

The diagnosis of (indolent) systemic mastocytosis (SM/ISM) was based on major and minor criteria [3,27]. The major criterion involves the presence of multifocal, dense aggregates of mast cells (≥15 mast cells per cluster) detected in bone marrow biopsies and/or in tissue sections from other extracutaneous organs. In addition, several minor criteria support the diagnosis: (1) at least 25% of mast cells in bone marrow smears show atypical morphology (type I or type II), or the mast cells are spindle-shaped within infiltrates found in bone marrow or other extracutaneous sites; (2) detection of an activating point mutation in the *KIT* gene, most commonly at codon 816, or in other critical regions of *KIT*, within bone marrow or extracutaneous tissue; (3) aberrant expression of CD2, CD25, and/or CD30 on mast cells in bone marrow, peripheral blood, or other extracutaneous locations; and (4) a baseline serum tryptase concentration persistently exceeding 20 ng/mL. A diagnosis of SM was confirmed when at least one major criterion and one minor criterion were met, or when three minor criteria were fulfilled in the absence of a major criterion.

The diagnosis of bone marrow mastocytosis (BMM) was established when neither B-finding nor skin lesions were detectable and the basal tryptase level was below 125 ng/mL [3,27].

Smoldering systemic mastocytosis (SSM) was diagnosed in patients who met the criteria for SM, exhibited two or three B-findings, and had no C-findings. Additionally, the diagnosis required the absence of clinical or pathological signs consistent with mast cell leukemia (MCL) or an associated hematologic neoplasm (AHN) [3,27].

The diagnosis of aggressive systemic mastocytosis (ASM) was established when the diagnostic criteria for SM were fulfilled, and there was clear evidence of SM-induced organ damage, defined as C-findings. Additionally, the diagnosis required the absence of any clinical or pathological signs suggestive of mast cell leukemia (MCL) or an associated hematologic neoplasm (AHN) [3,27].

MCL was diagnosed when at least 20% of mast cells were present in the bone marrow aspirate [3,27].

In cases where the diagnostic criteria for SM were not fulfilled, classification was based on prior diagnosis and *KIT* mutation status. If the patient had a prior working diagnosis of monomorphic maculopapular cutaneous mastocytosis (MPCM) and tested positive for a *KIT* mutation, the diagnosis was defined as probable indolent systemic mastocytosis (PISM), with the assumption that the bone marrow biopsy result may have been falsely negative. Conversely, if the patient had a working diagnosis of MPCM but was negative for *KIT* mutations, the diagnosis remained monomorphic MPCM, with no evidence of systemic involvement [3,27].

In this study, all patients with MPCM had monomorphic MPCM or PISM.

### 2.4. Evaluation of the Respiratory Function

To evaluate pulmonary involvement, spirometry and the DLCO tests were performed in every patient enrolled in the study.

In line with ATS and ERS recommendations, obstructive ventilatory disorders were diagnosed when the Tiffeneau index (FEV_1_/FVC) was below the lower limit of normal (LLN), defined as below the 5th percentile. The severity of obstruction was classified based on FEV_1_ as a percentage of the predicted value: ≥70% of the average value—mild; 60–69% of the average value—moderate; 50–59% of the average value—moderate–severe; 35–49% of the average value—severe; and <35% of the average value—very severe bronchoconstriction.

An MEF_50_ <60% of the predicted value with a normal FEV_1_/FVC ratio indicated small airway dysfunction.

Impaired diffusion was defined as DLCO/VA below the LLN (5th percentile). The severity of diffusion impairment was categorized as: >60% of the average value—mild; 40–60% of the average value—moderate; and <40% of the average value—severe impairment of diffusion [24].

Patients with impaired DLCO underwent HRCT of the chest. The scans were performed using volumetric acquisition with a slice thickness of 1.0 mm and a high spatial frequency reconstruction algorithm. Scans were acquired in the supine position during full inspiration. When indicated, additional expiratory phase imaging was obtained to evaluate for air trapping. The scans were typically performed without intravenous contrast, using 120 kVp and automated mA modulation. Images were reconstructed with both lung and mediastinal windows for comprehensive assessment of parenchymal and airway abnormalities.

### 2.5. Statistical Analysis

Categorical variables were described using frequencies and percentages. Associations between groups were evaluated using contingency tables and the Chi-square test, with Yates’ correction applied when appropriate.

Continuous variables were presented as medians with 95% confidence intervals (CIs) or means with standard deviation. The Shapiro–Wilk test was used to assess the normality of distributions. Based on distribution characteristics, group comparisons were conducted using either parametric methods (Student’s t-test or analysis of variance) or non-parametric alternatives (Mann–Whitney U test or Kruskal–Wallis test).

Moreover, a multivariate linear regression was performed to adjust for confounding variables (demographic data) to investigate whether the diagnosis of various types of mastocytosis influences pulmonary function.

All statistical analyses were conducted using Statistica 10 (StatSoft, Poland) and Python 3.10, incorporating the Pandas (v2.1.3) and Scikit-learn (v1.2.1) libraries. A two-sided *p*-value of less than 0.05 was considered statistically significant.

## 3. Results

### 3.1. Patients with Mastocytosis in Comparison to Healthy Controls

A total of 104 Caucasian patients with mastocytosis and mast cell activation syndrome were studied. The control group consisted of 71 healthy participants. Patients with mastocytosis were significantly older (median age 46 years; 95% CI 44.1–49.0; 34.6% males; Table 1) compared to the control group (median age 39 years; 95% CI 38.2–45.0; 50.7% males). Moreover, 42.3% of patients had a history of smoking. Therefore, multivariate linear regression was employed to adjust for potential confounders (age, sex, height, body mass, smoking history) when analyzing the relationship between mastocytosis and pulmonary function in comparison to controls.

In multivariate analysis, patients with mastocytosis showed no statistically significant differences in absolute FEV_1_ values (3.1 vs. 3.5 L; *p* = 0.054) or FEV_1_ as a percentage of the predicted value (1.0 vs. 1.0; *p* = 0.151).

However, significant differences were observed in several pulmonary function parameters. Compared to the control group (79.5 [95% CI: 76.7–79.6]), the FEV_1_/VC MAX ratio was lower in patients with mastocytosis (73.7 [95% CI: 71.8–75.0]). In comparison with the controls, DLCO/VA (mL/min/mmHg/L) was lower in the mastocytosis group (6.0 [95% CI: 5.9–6.5] vs. 1.4 [95% CI: 1.4–1.5]), as was the predicted value of DLCO/VA (1.003 [95% CI: 0.986–1.009] vs. 0.895 [95% CI: 0.876–0.926]). Compared to the controls, DLCOcSB (mL/min/mmHg) was also lower in patients with mastocytosis (9.5 [95% CI: 9.2–10.1] vs. 7.6 [95% CI: 7.5–8.2]), as was the DLCOcSB predicted value (0.994 [95% CI: 0.982–1.030] vs. 0.831 [95% CI: 0.825–0.875]). All differences in these parameters were statistically significant (*p* < 0.001).

### 3.2. Clinical Manifestation of Mastocytosis

The study analyzed 65 patients with ISM, 13 with MPCM, 9 with PISM, 9 with BMM, 7 with ASM/MCL, and 1 with SSM (Table 2). The median serum basal tryptase level was significantly higher in the group of patients with aggressive types of mastocytosis (ASM/MCL 71.5 µg/mL and SSM 206.0 µg/mL) compared to patients with ISM (32.7 µg/mL), BMM (30.4 µg/mL), PISM (17.7 µg/mL), and MPCM (12.9 µg/mL, *p* < 0.001).

Approximately 85% of patients with ISM and BMM, 70% of those with ASM/MCL, and 100% of patients with SSM and PISM harbored the KIT D816V mutation. All patients with MPCM, SSM, and PISM demonstrated skin involvement, compared to 98% of those with ISM and 71% with ASM/MCL (*p* < 0.001). Darier’s sign was observed in 100% of patients with SSM, 92% with MPCM, 81% with ISM, 78% with PISM, and 57% with ASM/MCL (*p* < 0.001). Itching was reported in 100% of patients with ASM/MCL and SSM, 92% with MPCM, 89% with PISM, 80% with ISM, and none in the BMM group (*p* < 0.001). In contrast, anaphylaxis occurred in 100% of patients with BMM, followed by 71% of those with ASM/MCL, and approximately 30% of patients with MPCM, PISM, and ISM (*p* = 0.002).

Allergy of any type was diagnosed in 86% of patients with ASM/MCL, 78% with BMM, approximately 35% with ISM and PISM, and 15% with MPCM (*p* = 0.009).

According to the diagnostic criteria, 100% of patients with ASM/MCL exhibited positive C-findings (*p* < 0.001). None of the patients presented with ascites, portal hypertension, or malnutrition.

### 3.3. Clinical Manifestations of Pulmonary Diseases and Assessment of Respiratory Function

The typical respiratory symptoms reported in the study group were as follows: dyspnea (38 patients, 36.5%), wheezing (10 patients, 9.6%), cough (8 patients, 7.6%), and chest pain or chest tightness (23 patients, 22.1%) (Table 3). The frequency of the reported symptoms was as follows: occasionally—8 patients; 1–2x per month—10 patients; 1–2x per week—3 patients; daily—2 patients. The frequency of symptoms did not differ between groups of patients diagnosed with various types of mastocytosis (*p* > 0.05). The diagnosis of asthma was confirmed in eight patients (*p* = 0.817 between clinical groups). There were no significant differences between the clinical forms of mastocytosis in parameters such as airway obstruction (*p* = 0.654), FEV1 as a percentage of the predicted value (*p* = 0.463), MEF75 (*p* = 0.550), MEF50 (*p* = 0.260), and MEF25 (*p* = 0.089; Table 3).

Among the studied patients, an obstruction, according to the ATS and ERS guidelines, was recognized in eight (7.7%) patients. They all had a mild obstruction according to the spirometry measurements (FEV1 relative to predicted value was between 0.846 and 0.981; median 0.911%). Considering the smoking history, three patients denied smoking, two had 0–5 pack-years, and three had >5 pack-years in their history. Among this group of patients, mastocytosis was diagnosed as follows: MPCM in one case and ISM in seven cases. Among nine patients with obstruction, a history of asthma was confirmed in two cases (25.0%), whereas among the patients without obstruction, a diagnosis of asthma had previously been established in six cases (6.2%, *p* = 0.222).

### 3.4. Impairment of CO Diffusion

An impaired/decreased DLCO/VA was recognized in five patients (4.8%): one patient with MPCM, three patients with ISM, and one patient with ASM. Except for the diagnostic criteria (DLCO changes), there were no statistically significant differences in lung function between patients with mastocytosis with and without CO diffusion impairment (Table 4). All patients with impaired DLCO had never smoked or had less than 5 pack-years of smoking. They denied taking medications resulting in side effects in the lung tissue, and they had never been exposed to damaging occupational factors. Moreover, none of them had a history of cardiac diseases. Furthermore, no pathological changes were confirmed in the HRCT results in all patients with impaired DLCO.

## 4. Discussion

In this retrospective, cross-sectional, single-center study involving 104 patients with mastocytosis and 71 healthy controls, the following findings were observed: (1) compared to healthy controls, patients with mastocytosis exhibited significantly lower values in FEV1/VC ratio, absolute DLCO/VA, predicted DLCO/VA, absolute DLCOcSB, and predicted DLCOcSB; (2) commonly reported respiratory symptoms among mastocytosis patients included dyspnea (36.5%), chest tightness (22.1%), and wheezing (9.6%). The prevalence of these symptoms did not differ significantly across the various clinical subtypes of mastocytosis; (3) airway obstruction was identified in 7.7% of patients; however, it appeared to be independent of the mastocytosis subtype; (4) a decreased DLCO/VA ratio was observed in 4.8% of patients, but HRCT did not reveal any radiological evidence of underlying lung disease.

### 4.1. Respiratory Symptoms

In the studied population, the most commonly reported respiratory symptoms among patients with mastocytosis were dyspnea (36.5%), chest tightness or pain (22.1%), and wheezing (9.6%; Table 2 and Table 3). However, using chest tightness as an example, fewer than 5% of patients reported experiencing such symptoms frequently (i.e., at least once per week). Thus, while many patients with mastocytosis likely experience pulmonary symptoms, their frequent occurrence appears to be relatively uncommon. This observation is consistent with previously published studies, mainly case reports, with [5,6,7,8,9,10], indicating that severe clinical and biological (histological or molecular) impairment of the lungs in mastocytosis is rare.

Based on our data, mastocytosis seems to be a risk factor for the occurrence and exacerbation of respiratory symptoms; however, the prevalence of these symptoms did not differ significantly among the various clinical subtypes of mastocytosis (Table 2 and Table 3).

### 4.2. Dysfunction of the Diffusing Capacity for Carbon Monoxide

DLCO was severely impaired in 4.8% of patients with mastocytosis. Moreover, in comparison with the controls, DLCO/VA (mL/min/mmHg/L) was lower in the mastocytosis group (6.0 vs. 1.4), as was the predicted value of DLCO/VA (1.003 vs. 0.895). DLCOcSB (mL/min/mmHg) was also lower in patients with mastocytosis (9.5 vs. 7.6), as was the DLCOcSB predicted value (0.994 vs. 0.831). All differences in these parameters were statistically significant (*p* < 0.001; Table 1).

These findings are clinically relevant, as the significantly reduced DLCO, DLCO/VA, and DLCOcSB values in patients with mastocytosis—despite severe impairment occurring in only 4.8% of cases—may reflect early and subclinical pulmonary involvement, including mast cell-mediated interstitial changes or pulmonary vascular abnormalities [17,18,19,28,29]. Reduced DLCO parameters, particularly when statistically significant compared to controls, suggest impaired alveolar–capillary gas exchange, which is a recognized early marker of pulmonary pathology even in the absence of overt radiographic or spirometric abnormalities [17,18,19,28,29]. In other chronic inflammatory and fibrotic diseases, such as idiopathic pulmonary fibrosis, similar reductions in DLCO have been associated with poorer prognosis and increased mortality, indicating that these changes in mastocytosis may also carry prognostic value [28,30]. Moreover, mast cell infiltration and mediator release are known to contribute to tissue remodeling and fibrosis, which could explain the observed impairment in diffusing capacity [17,28,29,30]. Importantly, early identification of such functional impairment should prompt further diagnostic evaluation (e.g., HRCT, echocardiography) to assess for pulmonary hypertension or interstitial lung disease, both of which may require changes in patient management. Moreover, in previous studies, when impairment of the lung barrier was diagnosed, HRCT revealed sometimes an area of ground-glass opacity and widening of interalveolar septa, as well as micronodular and cystic changes and enlargement of lymph nodes [5,6,7,8,9,10]. The infiltration of mast cells in the lung tissue has been demonstrated using lung tissue biopsies [5]. Although HRCT revealed no abnormalities in our study, the literature data may help explain the observed reduction in respiratory performance among the studied cohort. Therefore, routine DLCO assessment in patients with mastocytosis may offer valuable insights into disease progression and guide clinical monitoring and therapeutic strategies.

Although symptoms are present, no macroscopic abnormalities are detected on HRCT in patients with reduced DLCO. Therefore, we speculate that the changes likely occur at the subcellular or biochemical level of the alveolar–capillary barrier [2,3,4,5,6,7,8,9]. These presumed subcellular alterations may manifest as decreased DLCO/VA ratios compared to the control group. However, molecular or cellular changes cannot be confirmed, as lung biopsies were not performed.

A decline in DLCO (single-breath method, DLCO-SB) is also associated with cumulative cigarette exposure, even among otherwise healthy individuals. Several studies have reported a significant reduction in DLCO in patients with higher pack-year histories [31,32]. However, in our cohort, none of the individuals with impaired diffusion capacity had more than 5 pack-years of smoking history, suggesting alternative contributing factors.

Mastocytosis is classified as a myeloproliferative disorder [1,2], and the observed impairment in DLCO may parallel pulmonary complications seen in hematologic malignancies. Wasielewska et al. demonstrated that reduced diffusion capacity can occur as a late effect of treatment for childhood leukaemia or Hodgkin lymphoma [33,34]. In our study, we observed a slightly higher incidence of DLCO impairment, which may reflect a disease-related mechanism or subclinical pulmonary involvement requiring further investigation.

We can only speculate about the pathogenesis of interstitial lung disease caused by SM. Kelly et al. found fibrosis in a lung biopsy [7], which may confirm that mast cells may be essential in pulmonary fibrosis through the activation of fibroblasts by histamine and the direct production of transforming growth factor β [29,35]. Despite this, it does not explain why some patients with SM develop interstitial lung disease.

### 4.3. Obstruction

In this study, airway obstruction was identified in 7.7% of patients. There were also no significant differences in the characteristics of mastocytosis patients considering the occurrence of obstruction. The described patients were of a similar age and had comparable BMIs, pack-years of smoking, and percentages of atypical mast cells in bone marrow biopsy assessments, CD2/CD25 expression, or bone marrow D816V mutation. The only statistically significant difference was noticed for lung parameters that are diagnostic for airway obstruction (FEV1/FVC; *p* < 0.001), which is an expected result in this group of patients.

Mochizuki et al. reported that in patients with uncontrolled asthma, histamine and tryptase levels in bronchoalveolar lavage (BAL) fluid correlated with bronchial hypersensitivity [8]. Airway obstruction is characterized by reduced airflow, which may result from airway narrowing—as seen in asthma or chronic bronchitis—or from loss of elastic recoil, as in emphysema [36].

Nevertheless, based on all our data and the aforementioned literature, we recommend that patients with mastocytosis should undergo systematic evaluation for pulmonary symptoms, with a thorough clinical interview that assesses both symptom frequency and impact on quality of life, as well as screening for and management of potential comorbidities.

### 4.4. Limitations

The retrospective nature of this study precludes the establishment of causal relationships. To more accurately assess the association between mast cell infiltration in the lungs and pulmonary symptoms, future prospective and longitudinal studies are warranted [37,38,39,40]. Moreover, validation of these findings in an independent patient cohort—ideally within a multicenter study—would enhance their generalizability. The study’s reliance on patient-reported symptoms and historical events introduces a risk of recall bias, potentially compromising data accuracy [37,38,39,40]. Additionally, the patient population was predominantly composed of individuals with MPCM and ISM. A more balanced representation of patients with MCAS and SSM/ASM/MCL would improve the statistical robustness of the analyses. Nonetheless, this study includes a relatively large sample size of 104 patients. Given that most prior investigations are limited to case reports [5,6,7,8,9,10], this study represents one of the first and largest cohort-based analyses exploring the relationship between mastocytosis and pulmonary involvement, thereby establishing a foundational reference for future research in this area.

Another limitation of the study is that bronchodilator reversibility testing was not performed. Given that the mastocytosis cohort was, on average, older—and age-related decline in lung function could influence spirometric values such as FEV_1_—assessing reversibility would have provided important context for interpreting the presence and nature of airflow limitation. Including this evaluation could have strengthened the conclusions regarding the role of mast cells in respiratory function. The absence of this assessment should be noted as a potential limitation.

## 5. Conclusions

Mastocytosis appears to be a risk factor for the onset and worsening of respiratory symptoms such as dyspnea, chest tightness, and wheezing. However, the prevalence of these symptoms did not differ significantly across the various clinical subtypes of mastocytosis. Moreover, airway obstruction and impairment of the alveolar–capillary membrane seem to occur independently of the clinical subtype of mastocytosis. Additionally, the causal relationship between pulmonary involvement, mast cell infiltration of the alveolar–capillary membrane, and the systemic circulation of mast cell mediators remains unclear and requires further research.

## Figures and Tables

**Table 1:** Comparison of patients with mastocytosis and healthy controls. Categorical variables were analyzed using counts and percentages. Continuous variables were summarized using medians and corresponding 95% CIs.

Variable	Mastocytosis	Controls	*p*-Value
Count	104 (59.4)	71 (40.6)	-
Male (%)	36.0 (34.6)	36.0 (50.7)	0.049
Age (years)	46.0 (44.1–49.0)	39.0 (38.2–45.0)	0.017
Height (cm)	169.0 (166.2–170.7)	170.0 (168.3–172.5)	0.416
Body mass (kg)	75.0 (73.3–80.9)	79.0 (76.3–86.1)	0.153
BMI (kg/m^2^)	25.7 (25.7–27.4)	25.8 (26.3–29.4)	0.495
Smoking history	44.0 (42.3)	0.0 (0.0)	<0.001
Multivariate linear regression			
FEV1 (l)	3.1 (3.1–3.4)	3.5 (3.2–3.6)	0.054
FEV1-predicted value	1.0 (1.0–1.1)	1.0 (0.987–1.0)	0.151
FEV1/VC MAX %	73.7 (71.8–75.0)	79.4 (76.9–79.8)	<0.001
DLCO/VA (mL/min/mmHg/L)	1.4 (1.4–1.5)	6.0 (5.9–6.5)	<0.001
DLCO/VA-predicted value	0.895 (0.876–0.926)	1.003 (0.986–1.009)	<0.001
DLCOcSB (mL/min/mmHg)	7.6 (7.5–8.2)	9.5 (9.2–10.1)	<0.001
DLCOcSB-predicted value	0.831 (0.824–0.876)	0.994 (0.982–1.030)	<0.001

**Table 2:** Symptoms of mastocytosis. Categorical variables were analyzed using counts and percentages. Continuous variables were summarized using medians and corresponding 95% CIs.

Variable	PISM	ISM	BMM	MPCM	MCL/ASM	SSM	*p*-Value
Count	9 (8.7)	65 (62.5)	9 (8.7)	13 (12.5)	7 (6.7)	1 (1.0)	-
Males (%)	3.0 (33.3)	22.0 (33.8)	6.0 (66.7)	3.0 (23.1)	2.0 (28.6)	0.0 (0.0)	0.356
Age	48.0 (42.9–59.8)	43.0 (40.9–46.7)	58.0 (46.4–62.9)	42.0 (35.6–56.8)	58.0 (47.9–63.8)	49.0 (49.0–49.0)	0.021
Height	160.0 (155.8–172.6)	169.0 (165.6–171.8)	172.0 (163.0–179.4)	169.0 (165.0–172.4)	168.0 (155.4–178.9)	169.0 (169.0–169.0)	0.765
Weight	69.0 (59.5–92.5)	72.0 (71.6–82.5)	83.0 (79.6–94.6)	76.0 (69.1–80.5)	64.0 (57.6–83.2)	80.0 (80.0–80.0)	0.224
BMI	28.4 (24.1–31.2)	25.1 (24.9–27.2)	29.4 (26.1–33.9)	26.3 (24.5–28.0)	25.5 (22.5–28.0)	28.0 (28.0–28.0)	0.223
Age-first symptoms	38.0 (33.1–43.8)	30.0 (26.7–32.0)	48.0 (40.0–56.2)	31.0 (21.6–43.0)	42.0 (31.7–51.7)	42.0 (42.0–42.0)	<0.001
Serum tryptase concentration	17.7 (12.6–26.1)	32.7 (36.7–56.7)	30.4 (24.4–53.9)	12.9 (9.4–17.0)	71.5 (14.1–271.0)	206.0 (206.0–206.0)	<0.001
Bone marrow CD2: +	3.0 (33.3)	53.0 (81.5)	7.0 (77.8)	5.0 (38.5)	4.0 (57.1)	0.0 (0.0)	<0.001
Bone marrow CD25: +	4.0 (44.4)	56.0 (86.2)	9.0 (100.0)	6.0 (46.2)	5.0 (71.4)	1.0 (100.0)	<0.001
Bone marrow histopathology mast cells: sparse	9.0 (100.0)	9.0 (13.8)	1.0 (11.1)	13.0 (100.0)	2.0 (28.6)	0.0 (0.0)	<0.001
Bone marrow histopathology mast cells: many	0.0 (0.0)	56.0 (86.2)	8.0 (88.9)	0.0 (0.0)	5.0 (71.4)	1.0 (100.0)	
Spindle-shaped mast cells in bone marrow: 0–1%	8.0 (88.9)	30.0 (46.2)	7.0 (77.8)	11.0 (84.6)	2.0 (28.6)	0.0 (0.0)	0.003
Spindle-shaped mast cells in bone marrow: 1–5	1.0 (11.1)	25.0 (38.5)	1.0 (11.1)	2.0 (15.4)	3.0 (42.9)	0.0 (0.0)	
Spindle-shaped mast cells in bone marrow: 5–10	0.0 (0.0)	7.0 (10.8)	0.0 (0.0)	0.0 (0.0)	1.0 (14.3)	0.0 (0.0)	
Spindle-shaped mast cells in bone marrow: >10	0.0 (0.0)	3.0 (4.6)	1.0 (11.1)	0.0 (0.0)	1.0 (14.3)	1.0 (100.0)	
Bone marrow D816V mutation	9.0 (100.0)	50.0 (76.9)	5.0 (55.6)	0.0 (0.0)	6.0 (85.7)	0.0 (0.0)	0.002
Major criterion	0.0 (0.0)	56.0 (86.2)	8.0 (88.9)	0.0 (0.0)	5.0 (71.4)	1.0 (100.0)	<0.001
Minor criterion: atypical morphology	0.0 (0.0)	1.0 (1.5)	1.0 (11.1)	0.0 (0.0)	0.0 (0.0)	0.0 (0.0)	0.458
Minor criterion: CD2/CD25 expression	4.0 (44.4)	58.0 (89.2)	9.0 (100.0)	6.0 (46.2)	5.0 (71.4)	1.0 (100.0)	<0.001
Minor criterion: KIT mutation (D816V)	9.0 (100.0)	50.0 (76.9)	5.0 (55.6)	0.0 (0.0)	6.0 (85.7)	0.0 (0.0)	<0.001
Minor criterion: elevated serum tryptase	2.0 (22.2)	46.0 (70.8)	8.0 (88.9)	2.0 (15.4)	7.0 (100.0)	1.0 (100.0)	<0.001
Overall SM criteria fulfilment	0.0 (0.0)	65.0 (100.0)	9.0 (100.0)	0.0 (0.0)	7.0 (100.0)	1.0 (100.0)	<0.001
Skin involvement	9.0 (100.0)	64.0 (98.5)	0.0 (0.0)	13.0 (100.0)	5.0 (71.4)	1.0 (100.0)	<0.001
Percentage area of skin involvement	65.0 (49.2–90.8)	81.0 (57.0–73.1)	0.0 (0.0–0.0)	90.0 (62.5–89.5)	45.0 (4.2–68.4)	90.0 (90.0–90.0)	<0.001
Darier’s sign	7.0 (77.8)	53.0 (81.5)	0.0 (0.0)	12.0 (92.3)	4.0 (57.1)	1.0 (100.0)	<0.001
Splenomegaly	0.0 (0.0)	0.0 (0.0)	0.0 (0.0)	0.0 (0.0)	3.0 (42.9)	0.0 (0.0)	<0.001
Hepatomegaly	0.0 (0.0)	0.0 (0.0)	0.0 (0.0)	0.0 (0.0)	2.0 (28.6)	1.0 (100.0)	<0.001
Lymphadenopathy	0.0 (0.0)	0.0 (0.0)	0.0 (0.0)	0.0 (0.0)	1.0 (14.3)	0.0 (0.0)	0.006
Osteoporosis	1.0 (11.1)	7.0 (10.8)	1.0 (11.1)	1.0 (7.7)	3.0 (42.9)	0.0 (0.0)	0.300
Osteopenia	1.0 (11.1)	16.0 (24.6)	1.0 (11.1)	3.0 (23.1)	3.0 (42.9)	0.0 (0.0)	0.658
Bone pain	5.0 (55.6)	15.0 (23.1)	4.0 (44.4)	2.0 (15.4)	4.0 (57.1)	0.0 (0.0)	0.900
Flushing	5.0 (55.6)	31.0 (47.7)	0.0 (0.0)	6.0 (46.2)	5.0 (71.4)	1.0 (100.0)	0.050
Itching	8.0 (88.9)	52.0 (80.0)	0.0 (0.0)	12.0 (92.3)	7.0 (100.0)	1.0 (100.0)	0.000
Angioedema	4.0 (44.4)	9.0 (13.8)	0.0 (0.0)	3.0 (23.1)	0.0 (0.0)	0.0 (0.0)	0.080
Recurring hypotension	4.0 (44.4)	14.0 (21.5)	0.0 (0.0)	4.0 (30.8)	3.0 (42.9)	0.0 (0.0)	0.323
Anaphylaxis	3.0 (33.3)	22.0 (33.8)	9.0 (100.0)	4.0 (30.8)	5.0 (71.4)	0.0 (0.0)	0.002
Diarrhea	2.0 (22.2)	13.0 (20.0)	1.0 (11.1)	3.0 (23.1)	4.0 (57.1)	0.0 (0.0)	0.322
Stomach pain	2.0 (22.2)	17.0 (26.2)	0.0 (0.0)	3.0 (23.1)	5.0 (71.4)	0.0 (0.0)	0.048
Headache	3.0 (33.3)	12.0 (18.5)	4.0 (44.4)	6.0 (46.2)	1.0 (14.3)	0.0 (0.0)	0.133
Allergy	3.0 (33.3)	25.0 (38.5)	7.0 (77.8)	2.0 (15.4)	6.0 (85.7)	0.0 (0.0)	0.009
Food allergy	2.0 (22.2)	4.0 (6.2)	1.0 (11.1)	0.0 (0.0)	3.0 (42.9)	0.0 (0.0)	0.024
Hymenoptera allergy	9.0 (100.0)	49.0 (75.4)	2.0 (22.2)	11.0 (84.6)	7.0 (100.0)	1.0 (100.0)	0.001
Drug allergy	3.0 (33.3)	12.0 (18.5)	2.0 (22.2)	2.0 (15.4)	2.0 (28.6)	0.0 (0.0)	0.869
B-findings	0.0 (0.0)	0.0 (0.0)	0.0 (0.0)	0.0 (0.0)	3.0 (42.9)	1.0 (100.0)	<0.001
C-findings	0.0 (0.0)	0.0 (0.0)	0.0 (0.0)	0.0 (0.0)	7.0 (100.0)	0.0 (0.0)	<0.001

**Table 3:** Clinical manifestations of pulmonary diseases and assessment of respiratory function. Categorical variables were analyzed using counts and percentages. Continuous variables were summarized using medians and corresponding 95% CIs.

Variable	PISM	ISM	BMM	MPCM	MCL/ASM	SSM	*p*-Value
Dyspnea	4.0 (44.4)	23.0 (35.4)	1.0 (11.1)	5.0 (38.5)	5.0 (71.4)	0.0 (0.0)	0.934
Cough	1.0 (11.1)	6.0 (9.2)	0.0 (0.0)	1.0 (7.7)	0.0 (0.0)	0.0 (0.0)	0.820
Wheezing	0.0 (0.0)	7.0 (10.8)	0.0 (0.0)	3.0 (23.1)	0.0 (0.0)	0.0 (0.0)	0.350
Asthma	0.0 (0.0)	6.0 (9.2)	0.0 (0.0)	1.0 (7.7)	1.0 (14.3)	0.0 (0.0)	0.817
Airway obstruction	0.0 (0.0)	7.0 (10.8)	0.0 (0.0)	1.0 (7.7)	0.0 (0.0)	0.0 (0.0)	0.654
Has ever smoked	3.0 (33.3)	26.0 (40.0)	4.0 (44.4)	8.0 (61.5)	2.0 (28.6)	1.0 (100.0)	0.503
FEV1	2.8 (2.5–3.6)	3.2 (3.1–3.5)	3.2 (2.5–3.9)	3.0 (2.6–3.6)	3.0 (1.9–4.2)	3.2 (3.2–3.2)	0.721
FEV1 relative to predicted value	1.1 (0.988–1.3)	1.0 (1.0–1.1)	0.948 (0.927–1.1)	1.0 (0.955–1.1)	1.1 (0.875–1.3)	1.1 (1.1–1.1)	0.463
FEV1/FVC	81.6 (74.4–85.2)	81.8 (78.7–81.9)	76.1 (72.1–80.7)	81.3 (75.0–83.4)	81.8 (76.3–86.4)	89.8 (89.8–89.8)	0.351
FEV1/VC MAX	80.6 (72.6–83.4)	79.8 (76.7–80.2)	72.5 (70.8–79.0)	77.6 (73.2–81.5)	79.8 (71.8–93.7)	88.9 (88.9–88.9)	0.251
TLC	5.6 (4.6–6.3)	5.6 (5.3–5.9)	6.3 (5.1–6.8)	5.5 (4.9–5.9)	5.0 (3.5–7.2)	5.0 (5.0–5.0)	0.891
TLC relative to predicted value	1.0 (0.926–1.1)	0.972 (0.952–1.0)	1.0 (0.899–1.0)	0.982 (0.919–1.0)	0.982 (0.803–1.1)	0.935 (0.935–0.935)	0.798
RV	1.8 (1.1–2.4)	1.6 (1.4–3.4)	1.8 (1.5–2.1)	1.5 (1.4–1.7)	1.6 (−6.534–21.2)	1.2 (1.2–1.2)	0.629
RV relative to predicted value	0.803 (0.714–1.0)	0.849 (0.834–0.917)	0.829 (0.755–0.946)	0.848 (0.792–0.894)	0.918 (0.785–1.0)	0.679 (0.679–0.679)	0.793
DLCOcSB	7.3 (5.9 - 9.2)	7.7 (7.6–8.5)	8.5 (7.2–9.4)	7.5 (6.7–8.8)	5.8 (4.5–9.0)	6.8 (6.8–6.8)	0.501
DLCOcSB relative to predicted value	0.857 (0.752–1.0)	0.817 (0.814–0.879)	0.944 (0.812–1.0)	0.858 (0.786–0.915)	0.734 (0.622–0.942)	0.777 (0.777–0.777)	0.458
DLCO/VA	1.4 (1.3–1.5)	1.4 (1.4–1.5)	1.4 (1.3–1.7)	1.6 (1.3–1.7)	1.3 (1.1–1.4)	1.4 (1.4–1.4)	0.321
DLCO/VA relative to predicted value	0.891 (0.761–1.0)	0.874 (0.864–0.927)	1.0 (0.914–1.0)	0.917 (0.858–0.989)	0.809 (0.689–0.988)	0.89 (0.89–0.89)	0.326
MEF 75 (%)	1.0 (0.922–1.3)	1.0 (0.943–1.1)	0.912 (0.796–1.2)	0.994 (0.834–1.1)	1.1 (0.913–1.1)	1.3 (1.3–1.3)	0.550
MEF 50 (%)	0.927 (0.66–1.1)	0.85 (0.824–0.954)	0.784 (0.594–1.0)	0.849 (0.688–0.995)	0.972 (0.643–1.2)	1.5 (1.5–1.5)	0.260
MEF 25 (%)	0.683 (0.436–0.838)	0.631 (0.594–0.719)	0.451 (0.307–0.73)	0.581 (0.455–0.732)	0.891 (0.396–1.2)	1.3 (1.3–1.3)	0.089
MEF 75/25 (%)	0.913 (0.611–1.0)	0.822 (0.754–0.875)	0.643 (0.493–0.901)	0.764 (0.611–0.87)	0.852 (0.53–1.1)	1.4 (1.4–1.4)	0.112
Chest pain—never	7.0 (77.8)	53.0 (81.5)	6.0 (66.7)	9.0 (69.2)	5.0 (71.4)	1.0 (100.0)	0.601
Chest pain—less than once a month	0.0 (0.0)	5.0 (7.7)	1.0 (11.1)	0.0 (0.0)	2.0 (28.6)	0.0 (0.0)	
Chest pain—every month	1.0 (11.1)	6.0 (9.2)	1.0 (11.1)	2.0 (15.4)	0.0 (0.0)	0.0 (0.0)	
Chest pain—every week	1.0 (11.1)	0.0 (0.0)	1.0 (11.1)	1.0 (7.7)	0.0 (0.0)	0.0 (0.0)	
Chest pain—every day	0.0 (0.0)	1.0 (1.5)	0.0 (0.0)	1.0 (7.7)	0.0 (0.0)	0.0 (0.0)	

**Table 4:** Impairment of CO diffusion. Categorical variables were analyzed using counts and percentages. Continuous variables were summarized using medians and corresponding 95% CIs.

Variable	Normal CO Diffusion	Impaired CO Diffusion	*p*-Value
Count	99 (95.2)	5 (4.8)	-
FEV1	3.1 (3.1–3.4)	3.3 (3.0–3.9)	0.258
FEV1 relative to predicted value	1.0 (1.0–1.1)	1.1 (0.997–1.2)	0.444
FEV1/FVC	81.2 (78.6–81.2)	83.6 (69.9–91.8)	0.698
FEV1/VC MAX	79.2 (76.8–79.7)	83.5 (67.7–91.0)	0.514
Airway obturation	7.0 (7.1)	1.0 (20.0)	0.843
TLC	5.5 (5.3–5.8)	5.9 (5.1–6.5)	0.667
TLC relative to predicted value	0.972 (0.951–0.996)	1.1 (0.985–1.2)	0.063
RV	1.6 (1.5–3.6)	1.6 (1.4–1.7)	0.790
RV relative to predicted value	0.844 (0.834–0.897)	0.918 (0.879–0.968)	0.158
DLCOcSB	7.7 (7.6–8.3)	6.6 (5.3–8.0)	0.135
DLCOcSB relative to predicted value	0.839 (0.831–0.883)	0.73 (0.603–0.822)	0.017
DLCO/VA	1.4 (1.4–1.5)	1.1 (1.0–1.2)	0.001
DLCO/VA relative to predicted value	0.902 (0.889–0.936)	0.656 (0.625–0.703)	<0.001
MEF 75 (%)	1.0 (0.961–1.0)	1.1 (0.697–1.3)	0.908
MEF 50 (%)	0.85 (0.829–0.938)	0.972 (0.556–1.2)	0.923
MEF 25 (%)	0.625 (0.589–0.698)	0.891 (0.427–1.1)	0.207
MEF 75/25 (%)	0.82 (0.748–0.85)	0.954 (0.536–1.2)	0.622

## Data Availability

The data presented in this study are available upon request from the corresponding author due to legal restrictions and data privacy.
